# Supplementary material for: Early alterations of large-scale brain networks temporal dynamics in young children with autism
Source: Commun Biol. 2021 Aug 16;4:968. doi: 10.1038/s42003-021-02494-3 (PMC8367954; doi:10.1038/s42003-021-02494-3)
Supplement: Supplementary file 2 — Reporting summary. [file 42003_2021_2494_MOESM2_ESM.pdf]

nature portfolio

Corresponding author(s): Holger Sperdin

Last updated by author(s): Jul 13, 2021

Reporting Summary

Nature Portfolio wishes to improve the reproducibility of the work that we publish. This form provides structure for consistency and transparency in reporting. For further information on Nature Portfolio policies, see our Editorial Policies and the Editorial Policy Checklist.

Statistics

For all statistical analyses, confirm that the following items are present in the figure legend, table legend, main text, or Methods section.

n/a	Confirmed	
☐	☒	The exact sample size (*n*) for each experimental group/condition, given as a discrete number and unit of measurement
☐	☒	A statement on whether measurements were taken from distinct samples or whether the same sample was measured repeatedly
☐	☒	The statistical test(s) used AND whether they are one- or two-sided<br>*Only common tests should be described solely by name; describe more complex techniques in the Methods section.*
☐	☒	A description of all covariates tested
☐	☒	A description of any assumptions or corrections, such as tests of normality and adjustment for multiple comparisons
☐	☒	A full description of the statistical parameters including central tendency (e.g. means) or other basic estimates (e.g. regression coefficient) AND variation (e.g. standard deviation) or associated estimates of uncertainty (e.g. confidence intervals)
☐	☒	For null hypothesis testing, the test statistic (e.g. *F*, *t*, *r*) with confidence intervals, effect sizes, degrees of freedom and *P* value noted<br>*Give P values as exact values whenever suitable.*
☒	☐	For Bayesian analysis, information on the choice of priors and Markov chain Monte Carlo settings
☒	☐	For hierarchical and complex designs, identification of the appropriate level for tests and full reporting of outcomes
☐	☒	Estimates of effect sizes (e.g. Cohen's *d*, Pearson's *r*), indicating how they were calculated

Our web collection on statistics for biologists contains articles on many of the points above.

Software and code

Policy information about availability of computer code

Data collection	The EEG was acquired with a Hydrocel Geodesic Sensor Net (HCGSN, Electrical Geodesics, USA) with 129 scalp electrodes at a sampling frequency of 1000Hz. On-line recording was band-pass filtered at 0–100Hz using the vertex as reference.
Data analysis	Data pre-processing and microstate analysis were done using Cartool (http://sites.google.com/site/cartoolcommunity/) and Matlab (Natick, MA).

For manuscripts utilizing custom algorithms or software that are central to the research but not yet described in published literature, software must be made available to editors and reviewers. We strongly encourage code deposition in a community repository (e.g. GitHub). See the Nature Portfolio guidelines for submitting code & software for further information.

Data

Policy information about availability of data

All manuscripts must include a data availability statement. This statement should provide the following information, where applicable:

- Accession codes, unique identifiers, or web links for publicly available datasets
- A description of any restrictions on data availability
- For clinical datasets or third party data, please ensure that the statement adheres to our policy

The data that support the findings of this study are available on reasonable request from the corresponding author [MS]. The data are not publicly available due to them containing information that could compromise research participant privacy/consent.

Field-specific reporting

Please select the one below that is the best fit for your research. If you are not sure, read the appropriate sections before making your selection.

☒ Life sciences ☐ Behavioural & social sciences ☐ Ecological, evolutionary & environmental sciences

For a reference copy of the document with all sections, see nature.com/documents/nr-reporting-summary-flat.pdf

Life sciences study design

All studies must disclose on these points even when the disclosure is negative.

Sample size	Description of the sample is provided in the Method, participants subsection. In total, 293 participants were recruited for the experiment. We did not manage to place the cap on 108 participants. The cap was placed on the heads of 185 participants (110 ASD and 75 TD). Out of those, 60 participants (44 ASD and 16 TD) were excluded due to having too many movement-related artifacts, noisy signal, lack of interest or having insufficient number of epochs for subsequent analysis. Our sample size is above range when compared to other experiment in the field that include very young participants. Post-hoc power analyses depending on the observed effect sizes were made to estimate the relationship between the sample size of our group and the observed statistical power. The bootstrapping procedure served to estimate the likelihood of finding the true result we observed in our full cohort from smaller sample sizes of participants.
Data exclusions	The cap was placed on the heads of 185 participants (110 ASD and 75 TD). Out of those, 60 participants (44 ASD and 16 TD) were excluded due to having too many movement-related artifacts, noisy signal, lack of interest or having insufficient number of epochs for subsequent analysis. This was to be expected given the sensory processing issues frequently reported in ASD children (Kojovic, 2019). In order to minimize gender bias and include an equivalent percentage (20 percent) of females in both groups, we excluded 12 females from the TD group. As a result, the final sample consisted of 113 participants: 66 toddlers and preschoolers with ASD (11 females; mean age 3.3 years ± 1.0, range 1.8-5.9) and 47 TD peers (8 females; mean age 3.3 years ± 1.2, range 1.8-5.8). Groups did not differ by age (p = 0.8838) or sex (p = 0.9999).
Replication	The experiment does not involve replication. The method section, participants subsection clearly outlines our data exclusion criteria based on too many movement-related artefacts, unrepairable noisy signal, lack of interest, or insufficient amounts of epochs available for subsequent EEG microstates analysis.
Randomization	This information does not apply to the current experiment as we compare EEG microstates in toddlers and preschoolers with ASD compared to their TD peers. Thus no randomization nor masking was used.
Blinding	This information does not apply to the current experiment as we compare EEG microstates in toddlers and preschoolers with ASD compared to their TD peers. Thus no randomization nor masking was used.

Reporting for specific materials, systems and methods

We require information from authors about some types of materials, experimental systems and methods used in many studies. Here, indicate whether each material, system or method listed is relevant to your study. If you are not sure if a list item applies to your research, read the appropriate section before selecting a response.

Materials & experimental systems

n/a	Involved in the study	
☒	☐	Antibodies
☒	☐	Eukaryotic cell lines
☒	☐	Palaeontology and archaeology
☒	☐	Animals and other organisms
☐	☒	Human research participants
☒	☐	Clinical data
☒	☐	Dual use research of concern

Methods

n/a	Involved in the study	
☒	☐	ChIP-seq
☒	☐	Flow cytometry
☒	☐	MRI-based neuroimaging

Human research participants

Policy information about studies involving human research participants

Population characteristics	The final sample consisted of 113 participants: 66 toddlers and preschoolers with ASD (11 females; mean age 3.3 years ± 1.0, range 1.8-5.9) and 47 TD peers (8 females; mean age 3.3 years ± 1.2, range 1.8-5.8). Groups did not differ by age (p = 0.8838) or sex (p =0.9999).
Recruitment	All participants were recruited as a part of the Geneva Autism Cohort, a longitudinal cohort of young children. For the control group, TD toddlers and preschoolers were recruited through announcements in the Geneva community.

Ethics oversight	This study was approved by the Local Research Committee, the Commission Centrale d'Ethique de Recherche (CCER) in Geneva, Switzerland, and written informed consent was obtained from all children's parents prior to inclusion in the study.

Note that full information on the approval of the study protocol must also be provided in the manuscript.
