# Supplementary material for: Early alterations of large-scale brain networks temporal dynamics in young children with autism
Source: Commun Biol. 2021 Aug 16;4:968. doi: 10.1038/s42003-021-02494-3 (PMC8367954; doi:10.1038/s42003-021-02494-3)
Supplement: Supplementary file 4 — Description of Additional Supplementary Files. [file 42003_2021_2494_MOESM4_ESM.pdf]

Description of Additional Supplementary Files

File name: Supplementary Data 1

Description: The numbers underlying the graphs in figure 3 supplied in an excel file format.
